# Supplementary material for: Genome-wide association study in 176,678 Europeans reveals genetic loci for tanning response to sun exposure
Source: Nat Commun. 2018 May 8;9:1684. doi: 10.1038/s41467-018-04086-y (PMC5940788; doi:10.1038/s41467-018-04086-y)
Supplement: Supplementary file 1 — Supplementary Information [file 41467_2018_4086_MOESM1_ESM.pdf]

Genome-wide association study in 176,678 Europeans reveals genetic loci for tanning response to sun exposure

Visconti *et al*

Supplementary Figures

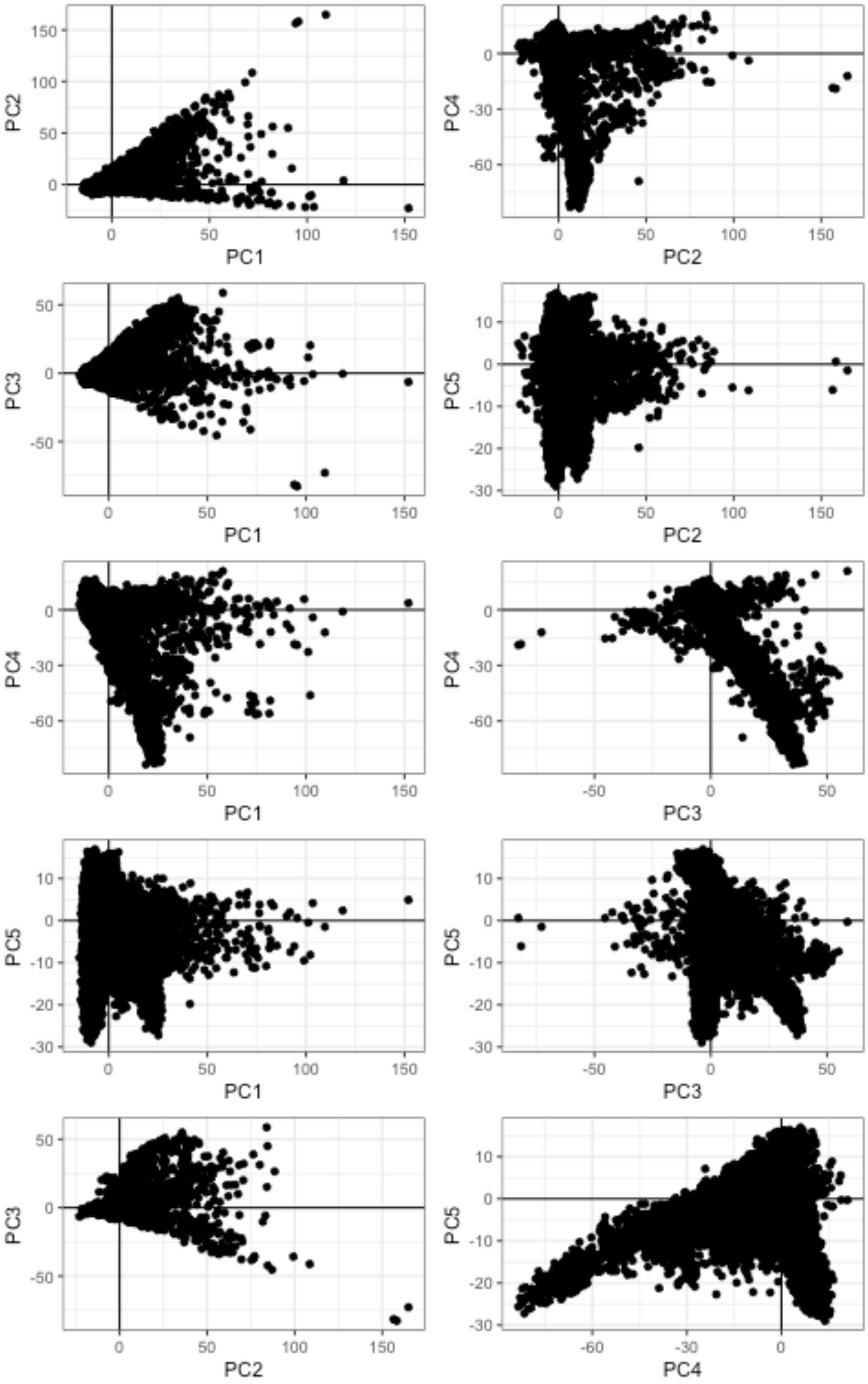

Supplementary Figure 1. Scatterplot of the first five principal components assessed on the UKBB genomic data. These principal components were added to the association model to control for potential stratification issues. Principal components were computed by the analysis group at the Wellcome Trust Centre for Human Genetics, University of Oxford. Details are provided at the UK Biobank website (http://biobank.ctsu.ox.ac.uk).

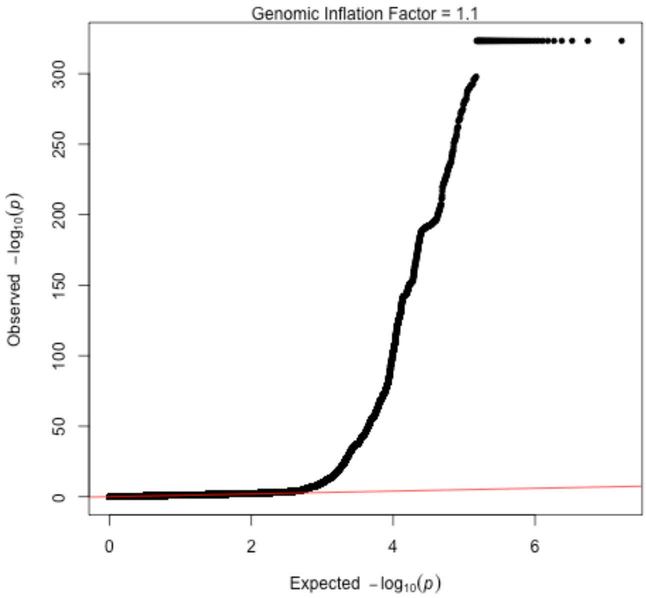

Supplementary Figure 2. Quantile-Quantile plot of observed *versus* expected P values for the 8,351,141 SNPs in the UKBB study. The P values were obtained by logistic regression analysis assuming additive model with sex and the first five principal components from the genotype data as covariates. P values are limited to $5x10^{-324}$ due to the minimum precision allowed by R and the *qqman* package.

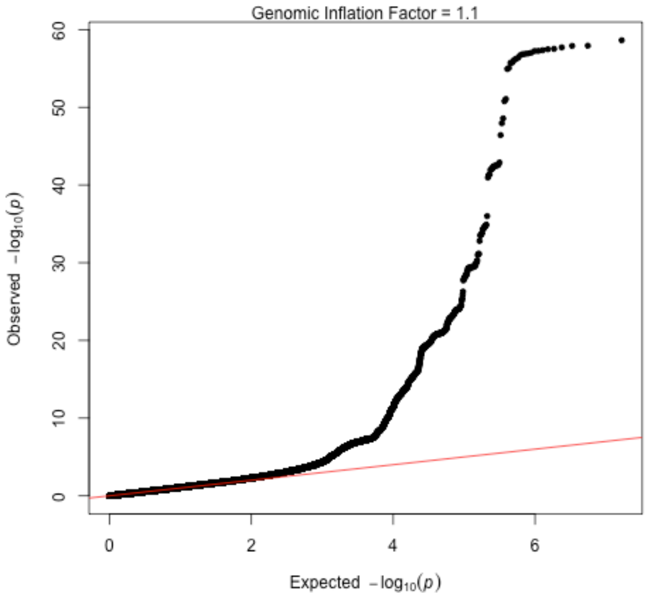

Supplementary Figure 3. Quantile-Quantile plot of observed *versus* expected P values after removing loci previously associated with ease of skin tanning. The P values were obtained by logistic regression analysis assuming additive model with sex and the first five principal components from the genotype data as covariates. The SNPs at the loci harbouring the genes *HERC2/OCA2, IRF4, MC1R, RALY/ASIP, SLC45A2,* and *TYR* were removed, resulting in 8,342,077 SNPs.

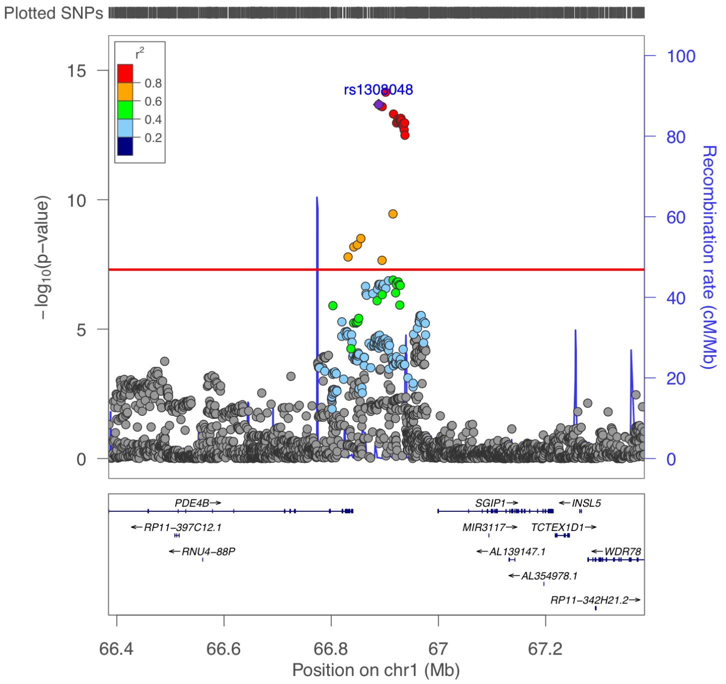

Supplementary Figure 4. Regional plot (chr1:66831370-66937516). Reference SNP is reported in blue. The horizontal red line shows the threshold of P=5x10^{-8}. LD information was evaluated on the genotype data from the UKBB cohort.

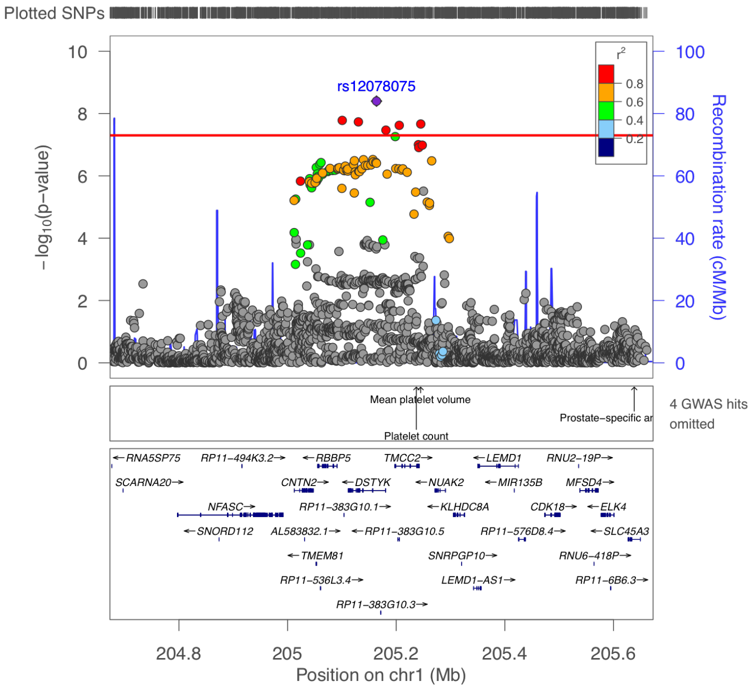

Supplementary Figure 5. Regional plot (chr1:205100663-205245233). Reference SNP is reported in blue. The horizontal red line shows the threshold of P=5x10^{-8}. LD information was evaluated on the genotype data from the UKBB cohort.

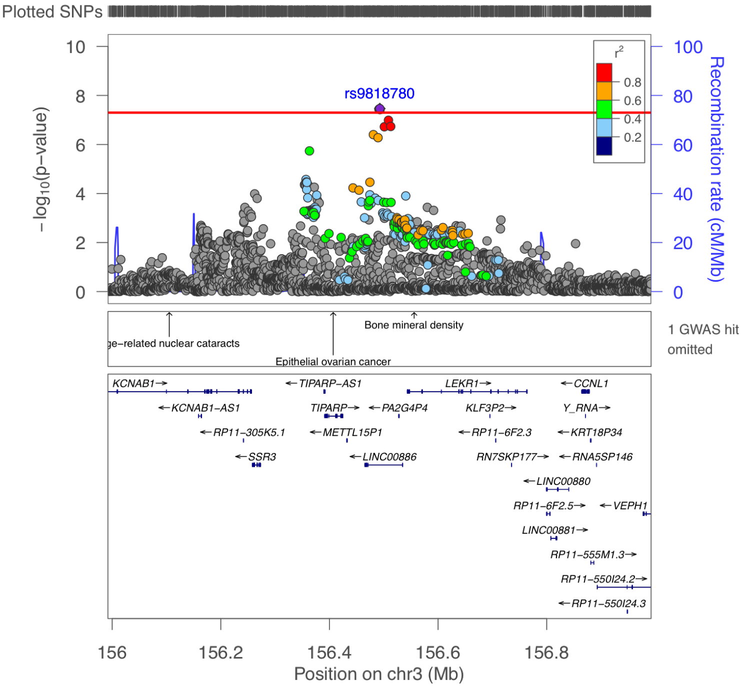

Supplementary Figure 6. Regional plot (chr3:156491160-156493213). Reference SNP is reported in blue. The horizontal red line shows the threshold of P=5x10^{-8}. LD information was evaluated on the genotype data from the UKBB cohort.

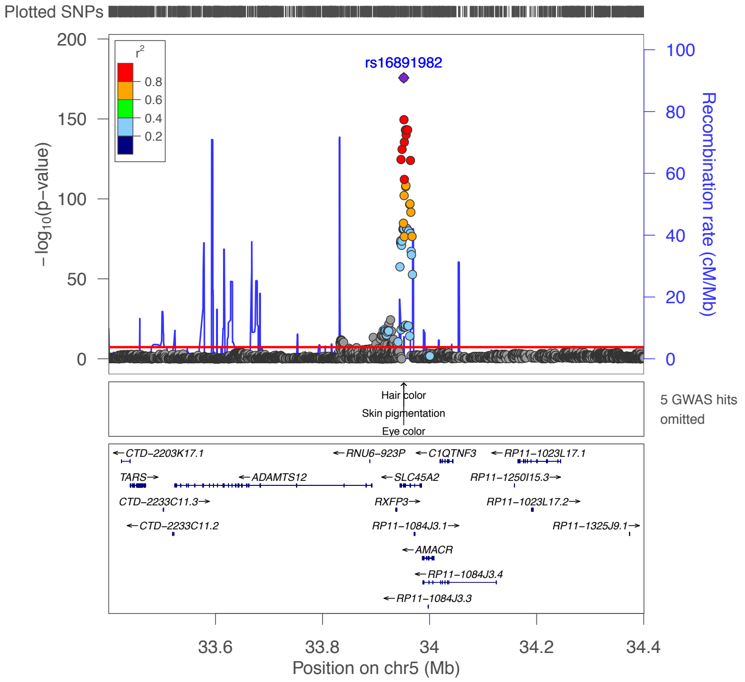

Supplementary Figure 7. Regional plot (chr5:33832958-33967955). Reference SNP is reported in blue. The horizontal red line shows the threshold of P=5x10^{-8}. LD information was evaluated on the genotype data from the UKBB cohort.

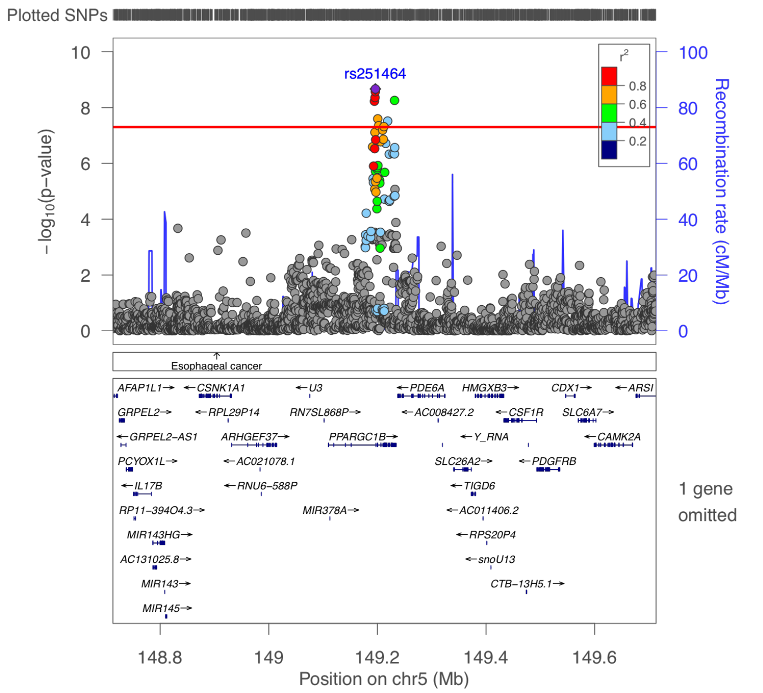

Supplementary Figure 8. Regional plot (chr5:149194485-149231519). Reference SNP is reported in blue. The horizontal red line shows the threshold of P=5x10^{-8}. LD information was evaluated on the genotype data from the UKBB cohort.

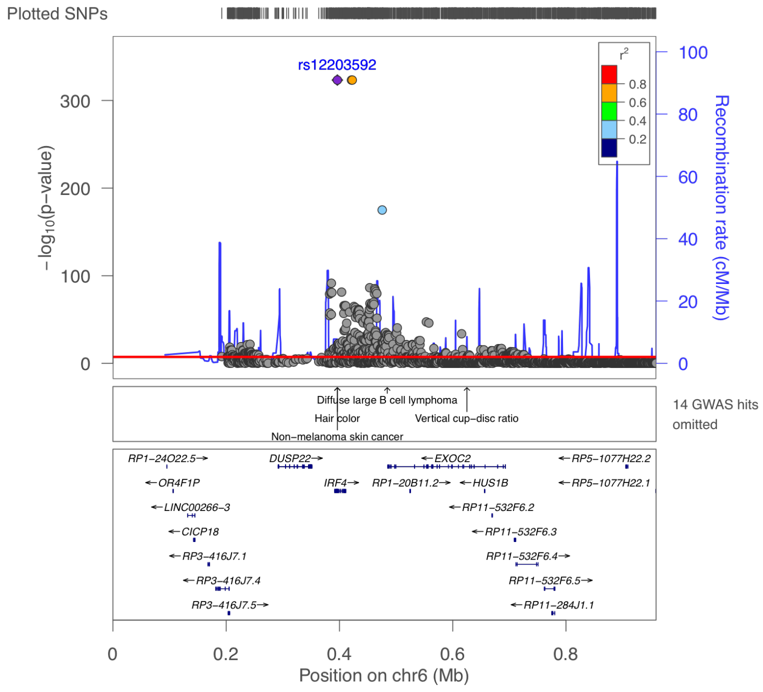

Supplementary Figure 9. Regional plot (chr6:192181-726042). Reference SNP is reported in blue. The horizontal red line shows the threshold of P=5x10^{-8}. LD information was evaluated on the genotype data from the UKBB cohort. P values are limited to 5x10^{-324} due to the minimum precision allowed by the *Locuszoom* software.

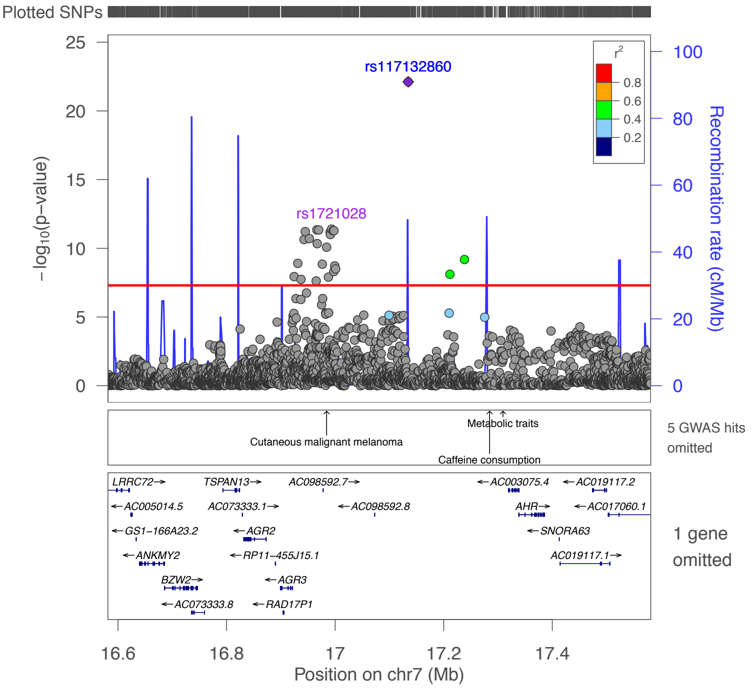

Supplementary Figure 10. Regional plot (chr7:16924528-17238316). Reference SNP is reported in blue; a further independent signal identified through conditional analysis is reported in violet. The horizontal red line shows the threshold of $P=5x10^{-8}$. LD information was evaluated on the genotype data from the UKBB cohort.

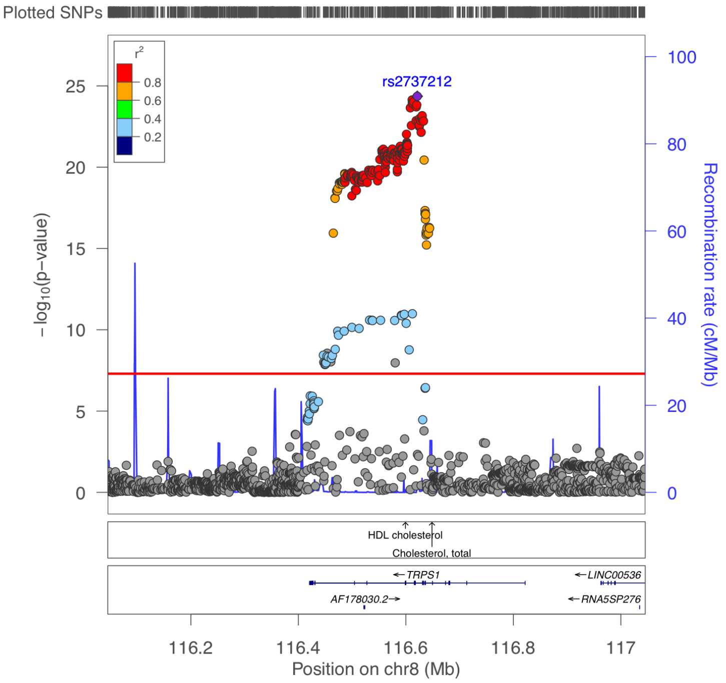

Supplementary Figure 11. Regional plot (chr8:116446547-116644121). Reference SNP is reported in blue. The horizontal red line shows the threshold of $P=5x10^{-8}$. LD information was evaluated on the genotype data from the UKBB cohort.

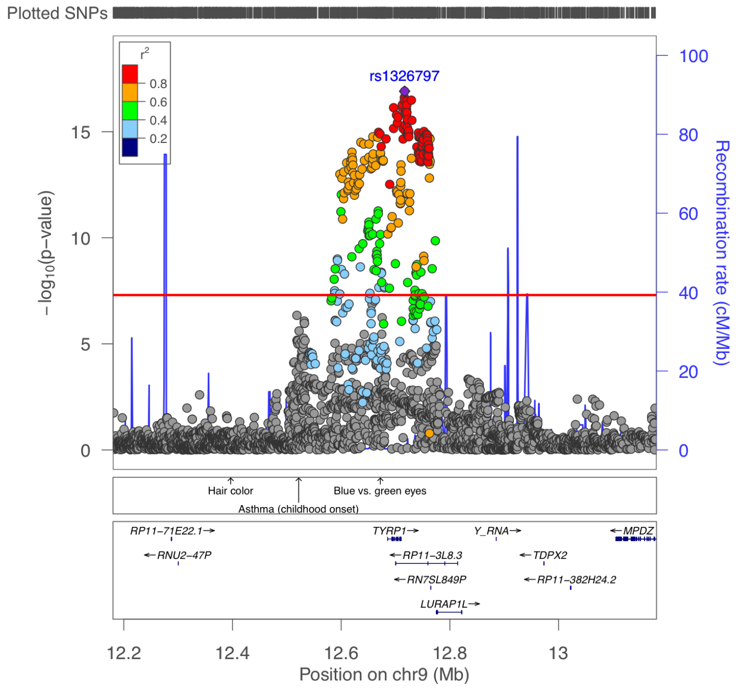

Supplementary Figure 12. Regional plot (chr9:12587153-12773263). Reference SNP is reported in blue. The horizontal red line shows the threshold of P= 5x10^{-8}. LD information was evaluated on the genotype data from the UKBB cohort.

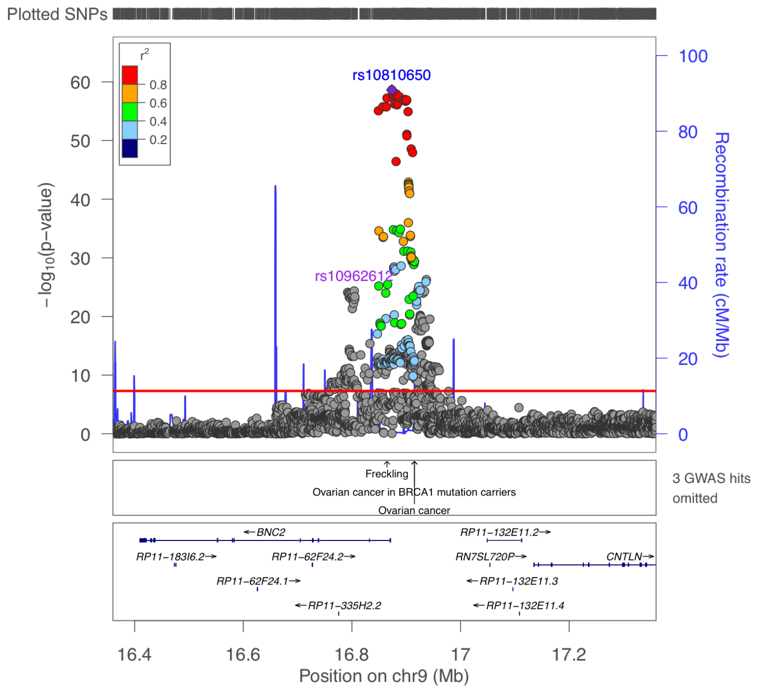

Supplementary Figure 13. Regional plot (chr9:16759161-16960741). Reference SNP is reported in blue, a further independent signal identified through conditional analysis is reported in violet. The horizontal red line shows the threshold of P=5x10^{-8}. LD information was evaluated on the genotype data from the UKBB cohort.

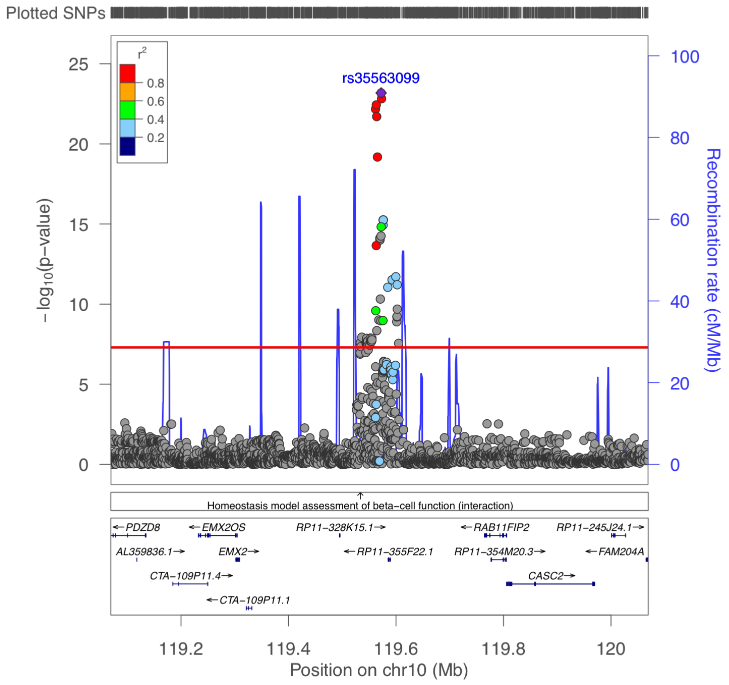

Supplementary Figure 14. Regional plot (chr10:119533757-119604938). Reference SNP is reported in blue. The horizontal red line shows the threshold of P=5x10^{-8}. LD information was evaluated on the genotype data from the UKBB cohort.

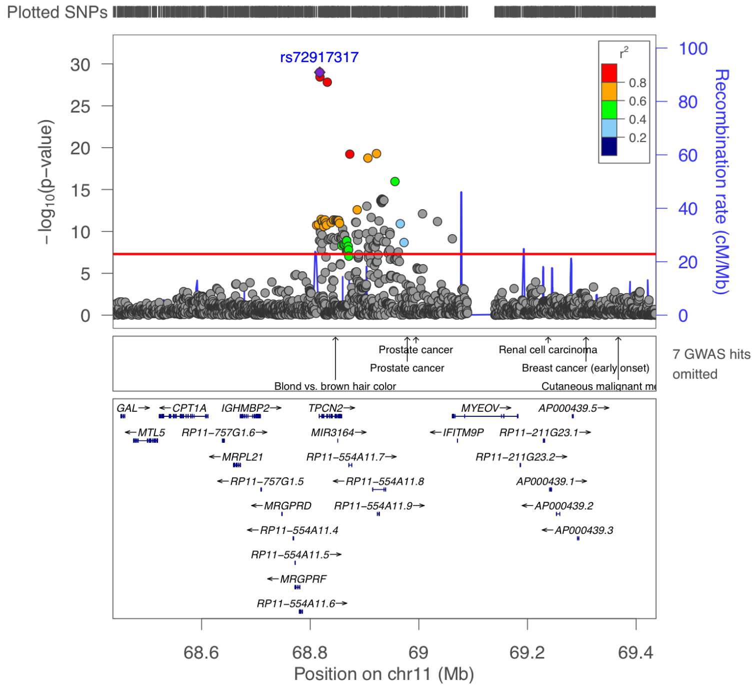

Supplementary Figure 15. Regional plot (chr11:68811777-69061635). Reference SNP is reported in blue. The horizontal red line shows the threshold of P=5x10^{-8}. LD information was evaluated on the genotype data from the UKBB cohort.

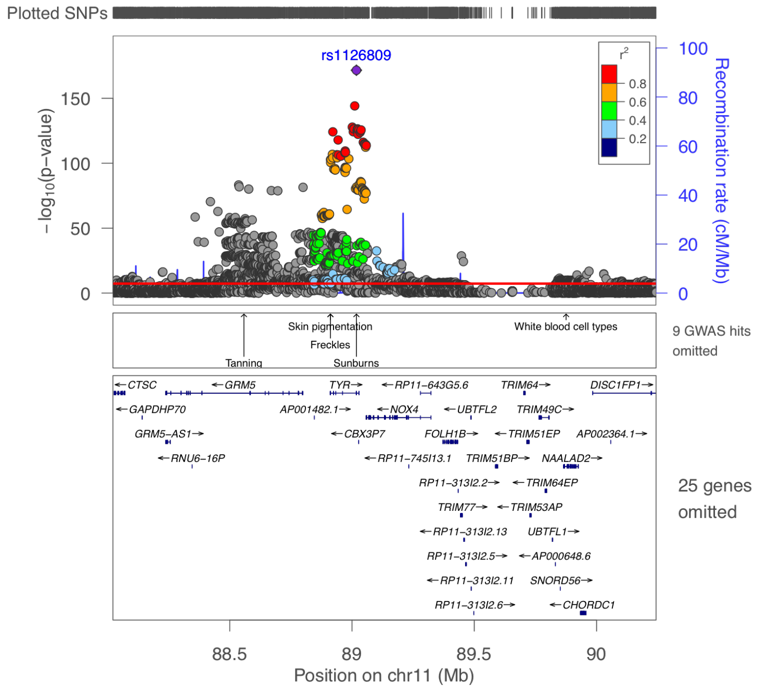

Supplementary Figure 16. Regional plot (chr11:88032224-90233385). Reference SNP is reported in blue. The horizontal red line shows the threshold of P=5x10^{-8}. LD information was evaluated on the genotype data from the UKBB cohort.

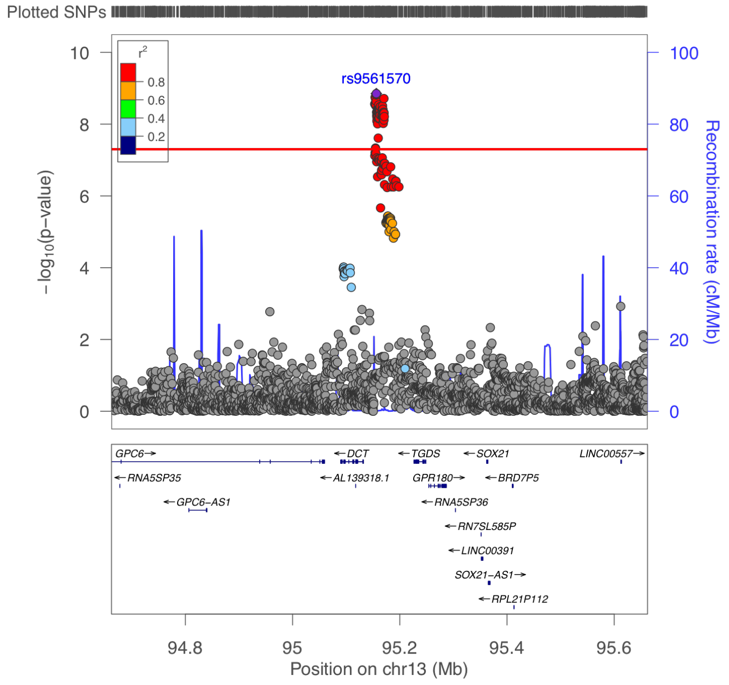

Supplementary Figure 17. Regional plot (chr13:95153167-95171058). Reference SNP is reported in blue. The horizontal red line shows the threshold of P=5x10^{-8}. LD information was evaluated on the genotype data from the UKBB cohort.

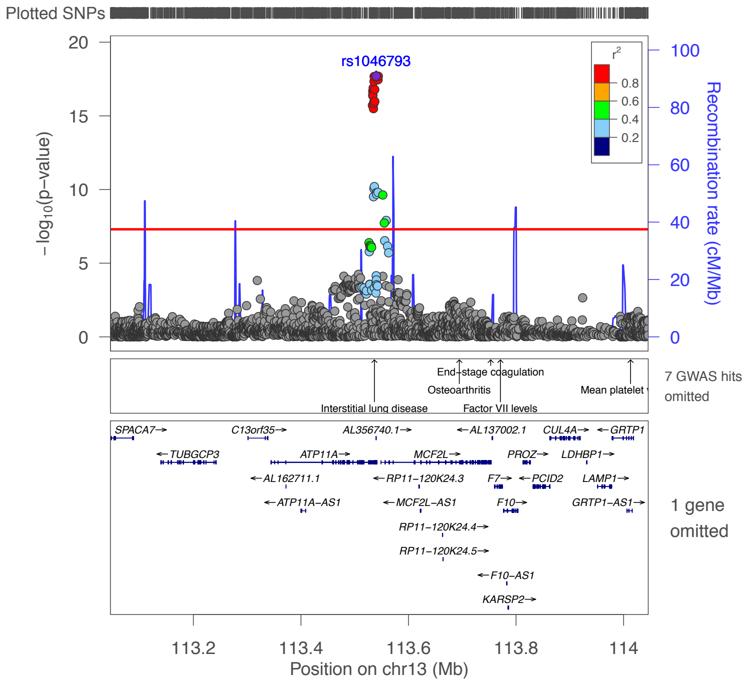

Supplementary Figure 18. Regional plot (chr13:113532990-113558599). Reference SNP is reported in blue. The horizontal red line shows the threshold of $P=5x10^{-8}$. LD information was evaluated on the genotype data from the UKBB cohort.

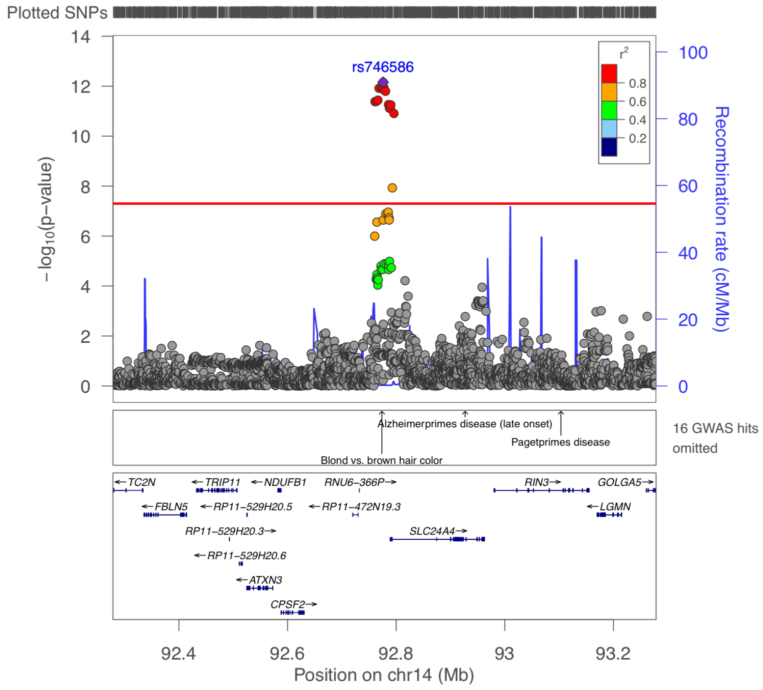

Supplementary Figure 19. Regional plot (chr14:92761113-92795912). Reference SNP is reported in blue. The horizontal red line shows the threshold of $P=5x10^{-8}$. LD information was evaluated on the genotype data from the UKBB cohort.

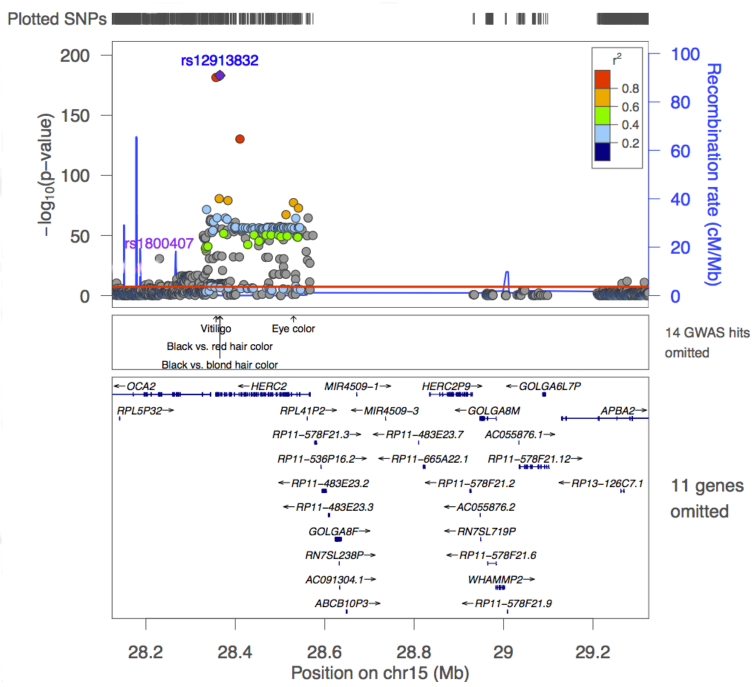

Supplementary Figure 20. Regional plot (chr15:28134352-29314924). Reference SNP is reported in blue, a further independent signal identified through conditional analysis is reported in violet. The horizontal red line shows the threshold of P=5x10^{-8}. LD information was evaluated on the genotype data from the UKBB cohort.

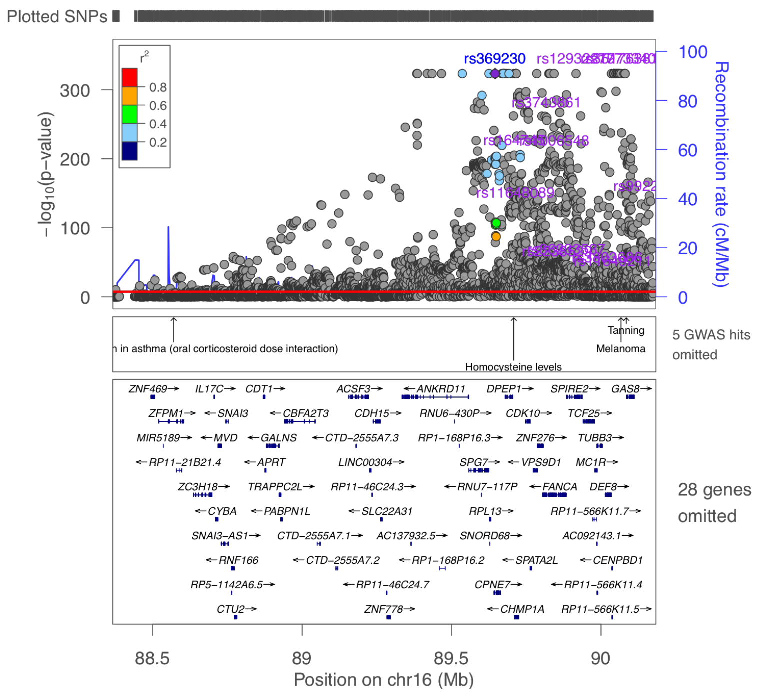

Supplementary Figure 21. Regional plot (chr16:88376014-90173553). Reference SNP is reported in blue, further independent signals identified through conditional analysis are reported in violet. The horizontal red line shows the threshold of P=5x10^{-8}. LD information was evaluated on the genotype data from the UKBB cohort. P values are limited to 5x10^{-324} due to the minimum precision allowed by the *Locuszoom* software.

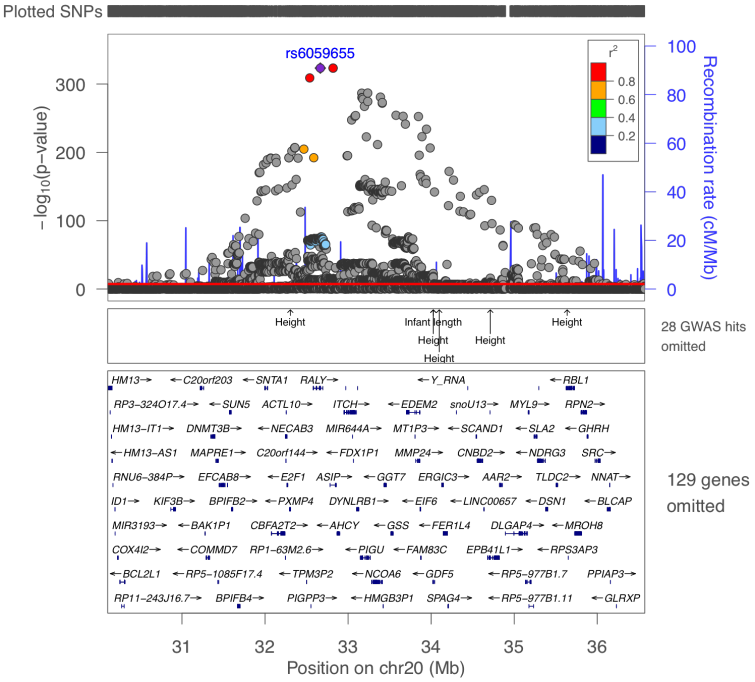

Supplementary Figure 22. Regional plot (chr20:30115523-36562529). Reference SNP is reported in blue. The horizontal red line shows the threshold of P=5x10^{-8}. LD information was evaluated on the genotype data from the UKBB cohort. P values are limited to 5x10^{-324} due to the minimum precision allowed by the *Locuszoom* software.

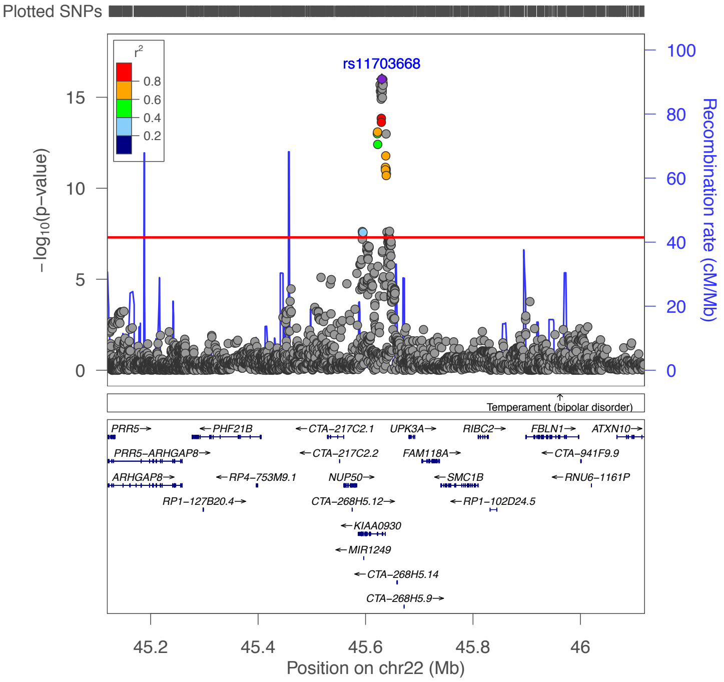

Supplementary Figure 23. Regional plot (chr22:45594002-45644654). Reference SNP is reported in blue. The horizontal red line shows the threshold of P=5x10^{-8}. LD information was evaluated on the genotype data from the UKBB cohort.

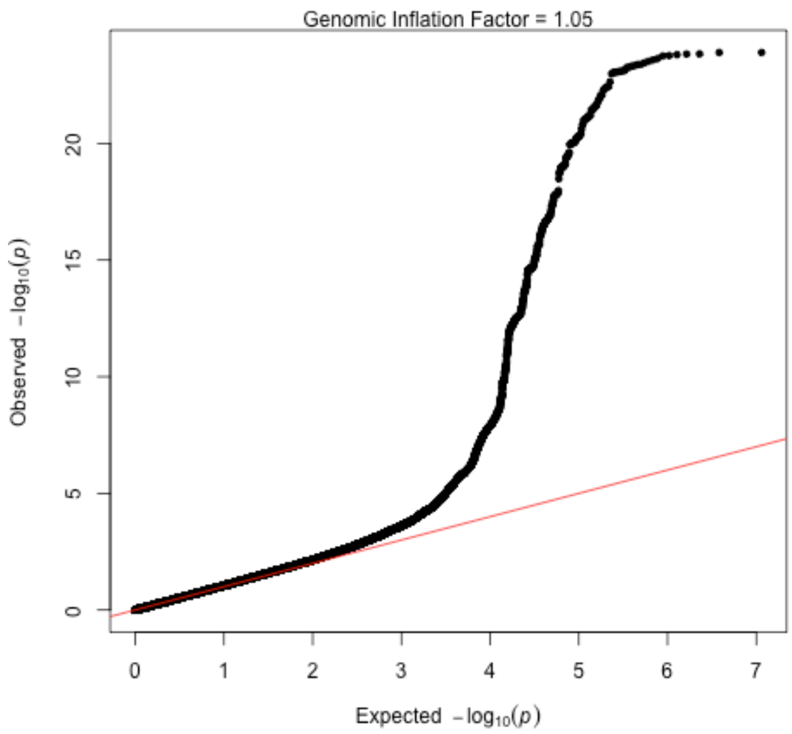

Supplementary Figure 24. Quantile-Quantile plot of observed *versus* expected P values for the 5,734,850 SNPs in the UKBB study for non-melanoma skin cancer. The P values were obtained by logistic regression analysis assuming additive model with age, sex, genotyping array, and the first five principal components from the genotype data as covariates.

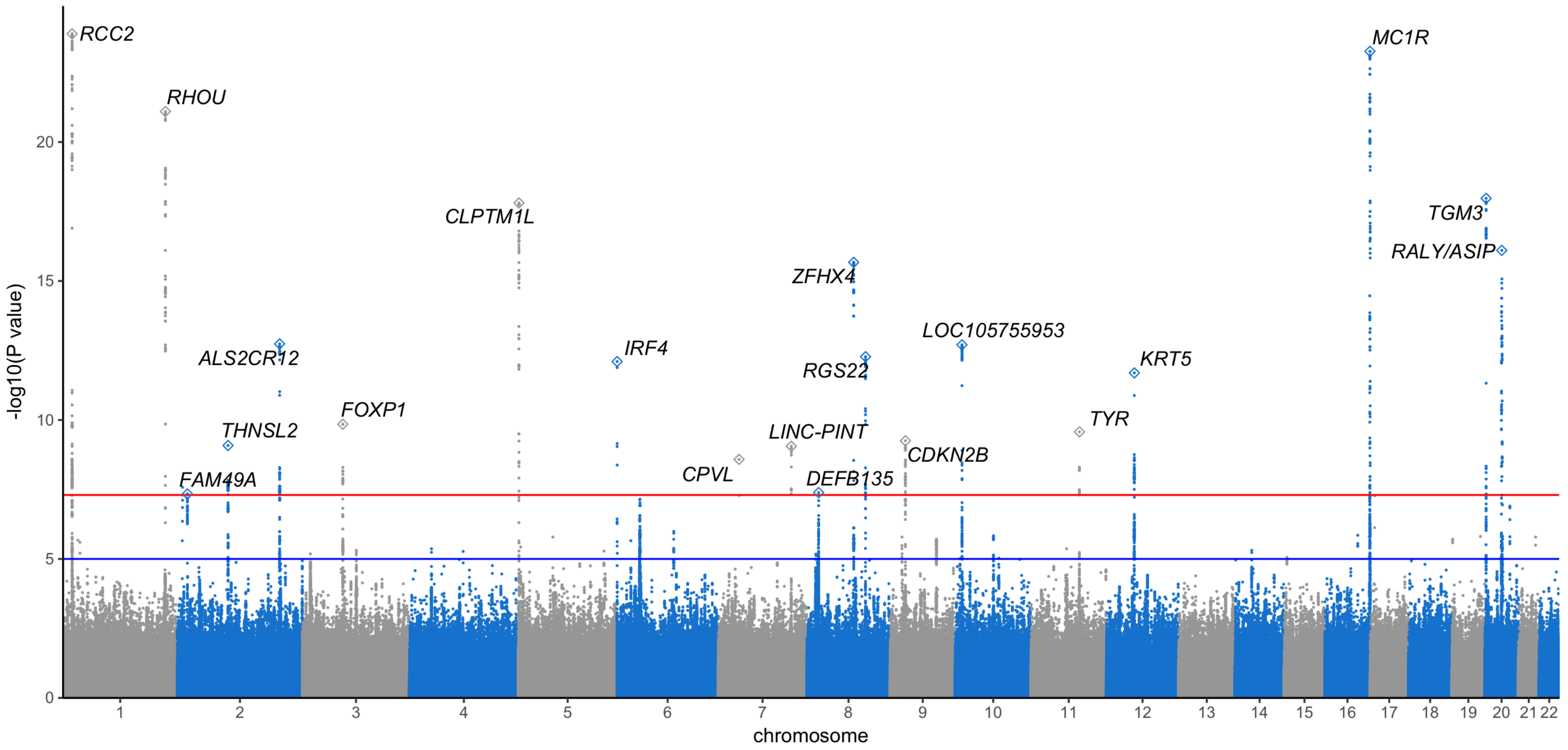

Supplementary Figure 25. Manhattan plot of non-melanoma skin cancer results in UKBB, second release. The P values were obtained by logistic regression analysis assuming an additive genetic model with age, sex, genotyping array, and the first five principal components of the genotype data as covariates. The x-axis shows the genomic coordinates (GRCh37.p13) of the tested SNPs and the y-axis shows the $-\log_{10}$ P value of their association. The horizontal red line indicates the threshold for genome-wide significance at 5.0×10^{-8}, the horizontal blue line indicates a relaxed threshold of 1.0×10^{-5}

Supplementary Tables

Supplementary Table 1. Phenotypic details of the individuals in the UKBB study sample.

	N	Mean Age	SD	First Quantile	Third Quantile
Low tan response					
All	46,768	56.58	8.12	50.00	63.00
Male	18,256	56.62	8.27	50.00	63.00
Female	28,512	56.56	8.02	50.00	63.00
High tan response					
All	74,528	57.09	7.99	51.00	64.00
Male	39,989	57.55	8.06	51.00	64.00
Female	34,539	56.55	7.88	50.00	63.00

Supplementary Table 2. **Summary of the 30 genetic loci identified in the UKBB sample.** For each locus, we reported the genomic coordinates (GRCh37.p13), the size of the region between the first and last genome-wide significant SNP ($P<5x10^{-8}$), the number of SNPs passing genome-wide significance within the region (N_G), and the number of independent signals (N_I) for which we sought replication. We also reported if the locus was replicated. * indicates that the locus only reached nominal significance in the replication step.

Coordinates	Size (bp)	N_G	N_I	Gene	Replicated
chr1:63727542-63727542	1	1	1	*FOXD3*	yes*
chr1:66831370-66937516	106147	26	1	*PDE4B*	yes
chr1:205100663-205245233	144571	6	1	*RIPK5*	yes
chr2:38166796-38321827	155032	50	1	*CYP1B1*	yes*
chr3:85439136-85640418	201283	160	1	*CADM2*	no
chr3:156491160-156493213	2054	3	1	*PA2G4P4*	yes
chr5:33832958-33967955	134998	106	1	*SLC45A2*	yes
chr5:59016897-59028853	11957	12	1	*PDE4D*	yes*
chr5:149194485-149231519	37035	9	1	*PPARGC1B*	yes
chr6:192181-726042	533862	519	1	*IRF4*	yes
chr7:16924528-17238316	313789	24	2	*AHR/AGR3*	yes
chr8:116446547-116644121	197575	232	1	*TRPS1*	yes
chr9:12587153-12773263	186111	223	1	*TYRP1*	yes
chr9:16759161-16960741	201581	467	3	*BNC2*	yes
chr10:119533757-119604938	71182	62	1	*EMX2*	yes
chr11:68811777-69061635	249859	122	1	*TPCN2*	yes
chr11:88032224-90233385	2201162	2103	2	*TYR*	yes
chr12:88406164-89422075	1015912	111	1	*KITLG*	yes*
chr13:95153167-95171058	17892	71	1	*DCT*	yes
chr13:113532990-113558599	25610	24	1	*ATP11A*	yes
chr14:92761113-92795912	34800	16	1	*SLC24A4*	yes
chr15:28134352-29314924	1180573	316	3	*HERC2/OCA2*	yes
chr15:29265171-29314924	49753	3	1	*APBA2*	no
chr15:48426484-48485926	59443	2	1	*SLC24A5*	no
chr16:71891862-71945613	53752	6	1	*IST1*	no
chr16:88376014-90173553	1797540	2983	19	*MC1R*	yes
chr20:25565515-25565515	1	1	1	*NINL*	no
chr20:30115523-36562529	6447007	3128	2	*RALY/ASIP*	yes
chr22:38551166-38552468	1303	2	1	*PLA2G6*	no
chr22:45594002-45644654	50653	43	1	*KIAA0930*	yes

Supplementary Table 3. Phenotypic details of the individuals in the TwinsUK sample. The TwinsUK sample used in this study includes only female individuals.

	N	Mean Age	SD	First Quantile	Third Quantile
Low tan response	1,651	49.07	12.60	41.10	58.50
High tan response	2,286	49.65	12.34	42.03	58.33

Supplementary Table 4. Phenotypic details of the individuals in the Rotterdam Study sample.

	N	Mean Age	SD	First Quantile	Third Quantile
Low tan response					
All	3,554	65.28	9.67	58.33	72.04
Male	1,390	64.12	8.91	58.03	69.98
Female	2,164	66.02	10.06	58.48	73.38
High tan response					
All	6,897	64.94	9.29	58.24	71.13
Male	3,049	64.36	8.71	58.10	70.16
Female	3,848	65.40	9.68	58.38	71.87

Supplementary Table 5. Phenotypic details of the individuals in the Queensland Institute of Medical Research (Brisbane Adolescent Twin Study, BATS) study sample: adolescent twins, siblings, and parents.

	N	Mean Age	SD	First Quantile	Third Quantile
Low tan response					
All	1,752	26.96	16.28	12.00	44.00
Male	816	26.45	16.88	12.00	44.00
Female	936	27.40	15.73	12.00	43.00
High tan response					
All	1,549	27.82	16.33	12.00	44.00
Male	766	27.42	16.89	12.00	45.00
Female	783	28.21	15.77	12.00	43.00

Supplementary Table 6. Phenotypic details of the individuals in the Queensland Institute of Medical Research (MARC7) study sample: adult twins.

	N	Mean Age	SD	First Quantile	Third Quantile
Low tan response					
All	1,093	52.27	8.11	46.30	57.70
Male	526	53.00	8.26	47.00	58.40
Female	567	51.59	7.91	45.40	57.30
High tan response					
All	755	52.40	7.93	46.30	57.90
Male	413	52.55	7.86	46.60	58.00
Female	342	52.21	8.03	46.10	57.60

Supplementary Table 7. Phenotypic details of the individuals in the NHS, NHS2, and HPFS studies.

	N	Mean Age	SD	First Quantile	Third Quantile
Low tan response					
All	7,497	45.26	10.4	37.00	52.00
Male	2,670	54.86	8.74	48.00	62.00
Female	4,827	39.95	6.83	35.00	44.00
High tan response					
All	28,348	44.64	9.73	37.00	51.00
Male	7,613	54.72	8.68	47.00	62.00
Female	20,735	40.94	7.12	35.00	47.00

Supplementary Table 8. Distribution of sex and ease of skin tanning in the five sub-studies of the NHS, NHS2, and HPFS cohorts.

Platform	Low tan response	High tan response	Male	Female	Total
Affymetrix	5,384	1,333	2,977	3,740	6,717
Illumina_HumanHap	4,492	1,101	1,100	4,493	5,593
OmniExpress	5,716	1,348	2,579	4,485	7,064
Oncoarray	7,317	1,914	2,101	7,130	9,231
HumanCoreExome	5,439	1,801	1,526	5,714	7,240
Total	28,348	7,497	10,283	25,562	35,845

Supplementary Table 9. Summary of associations replicated at a nominal significance. For each SNP, we report the genomic coordinates (GRCh37.p13), the effect allele, the minor allele frequency (MAF), the odds ratio (OR) along with its 95% confidence interval (CI) and standard error (SE), the association P value in the discovery set (P_{UKBB}), and the meta-analysis P values in the five independent replication cohorts ($P_{replication}$). Positive odds ratios indicate a decreased tanning ability. $^{+}$ indicates that the SNP is a secondary signal detected through conditional analysis.

SNP	CHR	BP	Effect Allele	MAF	OR (95% CI)	SE	P_{UKBB}	$P_{replication}$	Gene
rs670318	1	63727542	T	0.05	1.13 (1.09-1.17)	0.02	4.58×10^{-10}	3.34×10^{-2}	*FOXD3*
rs336024	2	38279469	C	0.19	1.07 (1.05-1.10)	0.01	1.19×10^{-10}	4.67×10^{-3}	*CYP1B1*
rs893191	5	59023325	T	0.38	1.05 (1.03-1.07)	0.01	1.50×10^{-8}	6.59×10^{-3}	*PDE4D*
rs1721028^{+}	7	16993426	C	0.42	1.06 (1.04-1.08)	0.01	4.02×10^{-12}	8.80×10^{-3}	*AGR3*
rs11104947	12	88942980	A	0.02	1.26 (1.18-1.35)	0.03	7.47×10^{-12}	1.40×10^{-2}	*KITLG*

Supplementary Table 10. Summary of independent associations detected through the conditional analysis. For each replicated independent signal identified in the conditional analysis, we report the genomic coordinates (GRCh37.p13), the effect allele, the minor allele frequency (MAF), the odds ratio (OR) along with its 95% confidence interval (CI) and standard error (SE), the association P value in the discovery set (P_{UKBB}), and the meta-analysis P values in the five independent replication cohorts ($P_{replication}$). Positive odds ratios indicate a decreased tanning ability.

SNP	CHR	BP	Effect Allele	MAF	OR (95% CI)	SE	P_{UKBB}	$P_{replication}$	Gene
rs10962612	9	16804167	T	0.24	0.90 (0.88-0.92)	0.01	4.49×10^{-25}	4.49×10^{-12}	*BCN2*
rs1800407	15	28230318	T	0.09	1.19 (1.16-1.23)	0.02	1.82×10^{-31}	1.58×10^{-6}	*OCA2*
rs164745	16	89709664	T	0.31	0.75 (0.73-0.76)	0.01	6.80×10^{-206}	3.50×10^{-42}	*CHMP1A*
rs11648089	16	89713938	C	0.11	0.70 (0.68-0.72)	0.02	9.54×10^{-130}	5.06×10^{-38}	*CHMP1A*
rs3743861	16	89818340	C	0.41	1.35 (1.33-1.37)	0.01	3.37×10^{-260}	4.14×10^{-57}	*FANCA*
rs1006548	16	89844043	C	0.22	0.72 (0.71-0.74)	0.01	4.18×10^{-205}	5.35×10^{-50}	*FANCA*
rs2238529	16	89853117	C	0.32	1.14 (1.12-1.16)	0.01	6.42×10^{-45}	4.35×10^{-7}	*FANCA*
rs36233537	16	89884127	G	0.03	0.66 (0.62-0.70)	0.03	2.28×10^{-47}	3.54×10^{-14}	*FANCA*
rs12932219	16	89916391	G	0.49	0.71 (0.70-0.72)	0.01	3.87×10^{-335}	2.09×10^{-86}	*SPIRE2*
rs3803686	16	90020346	C	0.19	0.87 (0.86-0.89)	0.01	1.82×10^{-35}	3.68×10^{-7}	*DEF8*
rs11649211	16	90039450	G	0.30	1.11 (1.09-1.13)	0.01	1.25×10^{-30}	3.60×10^{-14}	*AFG3L1P*
rs35176381	16	90062479	G	0.37	1.55 (1.52-1.57)	0.01	4.81×10^{-512}	1.05×10^{-139}	*AFG3L1P*
rs77733403	16	90080723	C	0.16	1.66 (1.62-1.70)	0.01	7.67×10^{-416}	2.61×10^{-82}	*DBNDD1*
rs9922277	16	90158838	C	0.36	1.25 (1.23-1.27)	0.01	3.72×10^{-139}	3.86×10^{-49}	*PRDM7*

Supplementary Table 11. Summary of the SNP-by-sex interaction models. For each SNP-by-sex interaction term significant in the UKBB study sample, we report the genomic coordinates (GRCh37.p13), the effect allele, the odds ratio (OR) along with its 95% confidence interval (CI) and standard error (SE), the association P value in the discovery set (P_{UKBB}), and the meta-analysis P values in the four independent replication cohorts ($P_{replication}$). Male was used as baseline in all the analysis. ⁺ indicates that the SNP is a secondary signal detected through conditional analysis

SNP	CHR	BP	Effect Allele	OR (95% CI)	SE	P_{UKBB}	$P_{replication}$
rs117132860	7	17134708	A	0.85 (0.76; 0.94)	0.05	1.73×10^{-3}	0.397
rs1800407⁺	15	28230318	T	0.90 (0.85; 0.95)	0.03	3.33×10^{-4}	0.052
rs369230	16	89645437	G	1.09 (1.05; 1.13)	0.02	3.59×10^{-6}	0.154
rs11648089⁺	16	89713938	C	0.90 (0.85; 0.95)	0.03	4.07×10^{-4}	0.338
rs1006548	16	89844043	C	0.93 (0.89; 0.97)	0.02	9.70×10^{-4}	0.897

Supplementary Table 12. CMM and non-melanoma skin cancer occurrence for the individuals in the UKBB study sample.

	N	Mean Age	SD	First Quantile	Third Quantile
CMM cases					
All	907	58.01	7.91	52.00	64.00
Male	372	58.80	7.76	53.00	65.00
Female	535	57.46	7.97	51.50	64.00
Non-melanoma skin cancer cases					
All	5,912	61.28	6.53	58.00	66.00
Male	3,035	61.97	6.26	59.00	67.00
Female	2,877	60.55	6.73	57.00	66.00
Controls					
All	181,740	56.34	8.14	50.00	63.00
Male	84,993	56.45	8.25	50.00	63.00
Female	96,747	56.24	8.03	50.00	63.00

Supplementary Table 13. Summary of associations with non-melanoma skin cancer. For each locus passing the genome-wide significant threshold ($P<5x10^{-8}$), we report its genomic coordinates (GRCh37.p13), the size of the region between the first and last genome-wide significant SNP, the number of SNPs passing genome-wide significance within the region (N_G), as well as the leading SNP, its effect allele, odds ratio (OR) and 95% confidence interval (CI), and standard error (SE). Positive OR represent an increased incidence of non-melanoma skin cancer. All loci but *CPVL, DEFB135, FAM49A, IRF4, LINC-PINT*, *THNSL2,* and *RALY/ASIP* were previously reported in Chahal *et al.,* 2016 (PMID:27539887). *IRF4*, and *RALY/ASIP* were previously reported in Chahal *et al.,* 2016 (PMID:27424798). *FAM49A* and *LINC-PINT* were previously reported in Stacey *et al.,* 2015 (PMID: 25855136).

Coordinate	Size (bp)	N_G	SNP	Effect Allele	OR (95% CI)	SE	P	Gene
chr1:17682100-17787980	105880	124	rs7528427	T	0.82 (0.79,0.85)	0.02	$1.27x10^{-24}$	*RCC2*
chr1:228941920-229021613	79693	60	rs12070203	T	0.82 (0.78,0.85)	0.02	$7.97x10^{-22}$	*RHOU*
chr2:7704860-7704860	1	1	rs79522206	A	0.75 (0.68,0.83)	0.05	$2.63x10^{-8}$	-
chr2:16499634-16499634	1	1	rs16982256	T	1.49 (1.29,1.73)	0.07	$4.59x10^{-8}$	*FAM49A*
chr2:88559607-88594573	34966	30	rs6709352	C	1.23 (1.15,1.31)	0.03	$8.26x10^{-10}$	*THNSL2*
chr2:202122995-202241907	118912	51	rs3769823	G	1.16 (1.12,1.21)	0.02	$1.83x10^{-13}$	*ALS2CR12*
chr3:71503479-71544614	41135	16	rs11707890	G	0.89 (0.85,0.92)	0.02	$1.423x10^{-10}$	*FOXP1*
chr5:1294086-1356771	62685	47	rs31487	C	1.18 (1.14,1.23)	0.02	$1.56x10^{-18}$	*CLPTM1L*
chr6:385735-434364	48629	5	rs147430042	G	0.58 (0.5,0.68)	0.07	$7.92x10^{-13}$	*IRF4*
chr7:29132279-29132279	1	1	rs117744081	G	1.45 (1.28,1.64	0.06	$2.62x10^{-9}$	*CPVL*
chr7:130581358-130585623	4265	9	rs157935	G	1.14 (1.09,1.19)	0.02	$8.80x10^{-10}$	*LINC-PINT*
chr8:11836318-1183631	1	1	rs11774568	A	0.89 (0.86,0.93)	0.02	$4.10x10^{-8}$	*DEFB135*
chr8:77437038-77499451	62413	33	rs17431641	T	1.45 (1.32,1.58)	0.04	$2.13x10^{-16}$	*ZFHX4*
chr8:100999637-101091569	91932	33	rs10099237	C	1.22 (1.16,1.29)	0.03	$5.28x10^{-13}$	*RGS22*
chr9:22017836-22056499	38663	26	rs10738605	G	1.13 (1.08,1.17)	0.02	$5.61x10^{-10}$	*CDKN2B*
chr10:8930198-9024785	94587	47	rs76141549	A	1.26 (1.19,1.34)	0.03	$1.98x10^{-13}$	*LOC105755953*
chr11:88912190-89058101	145911	11	rs3900053	C	0.88 (0.85,0.92)	0.02	$2.68x10^{-10}$	*TYR*
chr12:52814230-52913668	99438	44	rs11170164	T	0.80 (0.75,0.85)	0.03	$2.03x10^{-12}$	*KRT5*
chr16:89083136-90122562	1039426	94	rs12925026	T	0.75 (0.71,0.79)	0.03	$5.47x10^{-24}$	*MC1R*
chr20:2224985-2304832	79847	29	rs6082600	C	0.80 (0.76,0.84)	0.02	$1.06x10^{-18}$	*TGM3*
chr20:31551101-34269391	2718290	57	rs6059655	G	1.27 (1.20,1.35)	0.03	$7.91x10^{-17}$	*RALY/ASIP*

Supplementary Table 14. Natural hair colour phenotype details for the individuals in the UKBB study sample.

	N	Mean Age	SD	First Quantile	Third Quantile
Red					
All	4,701	56.55	8.12	50.00	63.00
Male	1,821	56.53	8.40	50.00	63.00
Female	2,880	56.56	7.92	50.00	63.00
Blonde					
All	13,433	56.8	8.07	50.00	63.00
Male	5,654	57.29	8.19	51.00	64.00
Female	7,779	56.44	7.96	50.00	63.00
Light Brown					
All	48,910	57.08	7.98	51.00	64.00
Male	22,501	57.64	8.05	52.00	64.00
Female	26,409	56.60	7.89	50.00	63.00
Dark					
All	51,733	56.66	8.08	50.00	63.00
Male	26,625	56.84	8.16	50.00	64.00
Female	25,108	56.46	8.00	50.00	63.00

Supplementary Table 15. Hair colour distribution according to ease of skin tanning for the individuals in the UKBB study sample.

	Red	Blonde	Light Brown	Dark
Low tan response				
All	4701	6786	19694	14464
Male	1821	2488	7513	5791
Female	2880	4298	12181	8673
High tan response				
All	0	6647	29216	37269
Male	0	3166	14988	20834
Female	0	3481	14228	16435

Supplementary Table 16. Summary of associations with non-red hair colour. For each replicated locus, we report the effect allele, the effect (BETA) along with its 95% confidence interval (CI) standard error (SE), and the association P value in the UKBB study sample for both non-red hair colour (HC) and tanning ability (TA). Hair colour was coded using a numerical value with blonde=1, light brown=2, and dark=3. The dark category included both individuals with dark brown and black hair. Positive $BETA_{HC}$ represent an increase in hair darkness; positive $BETA_{TA}$ represent a decreased tanning ability.

SNP	Effect	N_{HC}	$BETA_{HC}$ (95% CI)	SE_{SC}	P_{SC}	$BETA_{TA}$ (95% CI)	SE_{TA}	P_{TA}	Gene
rs1308048	C	111956	0.001 (-0.005; 0.006)	0.00	0.795	-0.068 (-0.021; -0.115)	-0.024	8.74×10^{-15}	*PDE4B*
rs12078075	G	113480	-0.050 (-0.060; -0.040)	0.00	3.20×10^{-23}	0.087 (0.016; 0.158)	0.036	1.03×10^{-8}	*RIPK5*
rs9818780	C	111207	-0.004 (-0.009; 0.002)	0.00	0.181	0.048 (0.007; 0.089)	0.021	4.44×10^{-8}	*PA2G4P4*
rs16891982	C	114076	0.299 (0.282; 0.316)	0.00	8.17×10^{-259}	-0.920 (-0.579; -1.261)	-0.174	1.19×10^{-173}	*SLC45A2*
rs251464	C	112039	0.002 (-0.005; 0.008)	0.00	0.619	-0.057 (-0.010; -0.104)	-0.024	1.39×10^{-8}	*PPARGC1B*
rs12203592	T	114076	0.254 (0.247; 0.260)	0.00	4.08×10^{-1180}	0.545 (0.396; 0.694)	0.076	1.91×10^{-567}	*IRF4*
rs117132860	A	114076	-0.015 (-0.032; 0.002)	0.00	0.092	0.263 (0.098; 0.428)	0.084	4.10×10^{-23}	*AHR/AGR3*
rs2737212	C	110542	-0.003 (-0.008; 0.003)	0.00	0.338	0.091 (0.036; 0.146)	0.028	6.98×10^{-25}	*TRPS1*
rs1326797	T	113143	0.035 (0.029; 0.040)	0.00	1.35×10^{-32}	-0.077 (-0.026; -0.128)	-0.026	5.75×10^{-18}	*TYRP1*
rs10810650	C	112034	0.026 (0.021; 0.032)	0.00	1.02×10^{-19}	-0.145 (-0.074; -0.216)	-0.036	3.15×10^{-59}	*BNC2*
rs35563099	T	109800	-0.005 (-0.013; 0.003)	0.00	0.207	-0.120 (-0.046; -0.194)	-0.038	5.41×10^{-23}	*EMX2*
rs72917317	G	109574	-0.132 (-0.141; -0.123)	0.00	3.92×10^{-166}	0.165 (0.069; 0.261)	0.049	7.64×10^{-30}	*TPCN2*
rs1126809	A	114076	-0.040 (-0.046; -0.034)	0.00	1.98×10^{-39}	0.255 (0.159; 0.351)	0.049	4.81×10^{-165}	*TYR*
rs9561570	T	113736	-0.036 (-0.042; -0.030)	0.00	5.34×10^{-33}	0.056 (0.011; 0.101)	0.023	1.19×10^{-9}	*DCT*
rs1046793	C	113818	0.005 (0.000; 0.011)	0.00	0.053	-0.076 (-0.025; -0.127)	-0.026	1.44×10^{-18}	*ATP11A*
rs746586	T	113220	-0.136 (-0.142; -0.130)	0.00	1.9×10^{-506}	0.062 (0.017; 0.107)	0.023	9.09×10^{-13}	*SLC24A4*
rs12913832	A	114076	0.369 (0.363; 0.375)	0.00	3.17×10^{-2736}	-0.307 (-0.195; -0.419)	-0.057	5.83×10^{-182}	*HERC2/OCA2*
rs369230	G	113329	-0.093 (-0.100; -0.087)	0.00	1.36×10^{-184}	0.466 (0.335; 0.597)	0.067	1.63×10^{-500}	*MC1R*
rs6059655	A	113302	-0.114 (-0.124; -0.105)	0.00	4.88×10^{-131}	0.527 (0.358; 0.696)	0.086	6.08×10^{-307}	*RALY/ASIP*
rs11703668	G	112405	0.013 (0.007; 0.019)	0.00	4.88×10^{-6}	-0.073 (-0.024; -0.122)	-0.025	4.76×10^{-17}	*KIAA0930*

Supplementary Table 17. Summary of associations with red versus non-red hair colour. For each replicated locus, we report the effect allele, the odds ratio (OR) along with its 95% confidence interval (CI) standard error (SE), and the association P value in the UKBB study sample for both red versus not-red hair colour (HC) and tanning ability (TA). Positive OR_{HC} represent an increased incidence of red hair; positive OR_{TA} represent a decreased tanning ability.

SNP	Effect Allele	N	OR_{HC} (95% CI)	SE_{HC}	P_{HC}	OR_{TA} (95% CI)	SE_{TA}	P_{TA}	Gene
rs1308048	C	116563	0.96 (0.92; 1.00)	0.02	0.070	0.93 (0.92; 0.95)	0.01	$8.74x10^{-15}$	*PDE4B*
rs12078075	G	118155	0.99 (0.92; 1.07)	0.04	0.786	1.09 (1.06; 1.12)	0.02	$1.03x10^{-8}$	*RIPK5*
rs9818780	C	115800	1.01 (0.97; 1.05)	0.02	0.629	1.05 (1.03; 1.07)	0.01	$4.44x10^{-8}$	*PA2G4P4*
rs16891982	C	118777	0.70 (0.60; 0.82)	0.08	$1.37x10^{-5}$	0.40 (0.37; 0.43)	0.03	$1.19x10^{-173}$	*SLC45A2*
rs251464	C	116648	0.98 (0.94; 1.03)	0.03	0.492	0.94 (0.93; 0.96)	0.01	$1.39x10^{-8}$	*PPARGC1B*
rs12203592	T	118777	1.27 (1.21; 1.33)	0.02	$1.61x10^{-22}$	1.72 (1.69; 1.76)	0.01	$1.91x10^{-567}$	*IRF4*
rs117132860	A	118777	1.03 (0.91; 1.17)	0.07	0.621	1.30 (1.24; 1.37)	0.03	$4.10x10^{-23}$	*AHR/AGR3*
rs2737212	C	115095	1.03 (0.98; 1.07)	0.02	0.232	1.09 (1.08; 1.11)	0.01	$6.98x10^{-25}$	*TRPS1*
rs1326797	T	117799	0.98 (0.94; 1.02)	0.02	0.283	0.93 (0.91; 0.94)	0.01	$5.75x10^{-18}$	*TYRP1*
rs10810650	C	116646	0.96 (0.92; 1.00)	0.02	0.056	0.86 (0.85; 0.88)	0.01	$3.15x10^{-59}$	*BNC2*
rs35563099	T	114312	1.01 (0.95; 1.07)	0.03	0.822	0.89 (0.87; 0.91)	0.01	$5.41x10^{-23}$	*EMX2*
rs72917317	G	114088	1.09 (1.02; 1.17)	0.04	0.014	1.18 (1.15; 1.21)	0.01	$7.64x10^{-30}$	*TPCN2*
rs1126809	A	118777	0.99 (0.95; 1.04)	0.02	0.798	1.29 (1.27; 1.31)	0.01	$4.81x10^{-165}$	*TYR*
rs9561570	T	118426	1.03 (0.99; 1.08)	0.02	0.141	1.06 (1.04; 1.08)	0.01	$1.19x10^{-9}$	*DCT*
rs1046793	C	118501	0.99 (0.95; 1.03)	0.02	0.550	0.93 (0.91; 0.94)	0.01	$1.44x10^{-18}$	*ATP11A*
rs746586	T	117885	0.98 (0.94; 1.02)	0.02	0.297	1.06 (1.05; 1.08)	0.01	$9.09x10^{-13}$	*SLC24A4*
rs12913832	A	118777	0.74 (0.70; 0.78)	0.03	$4.23x10^{-27}$	0.74 (0.72; 0.75)	0.01	$5.83x10^{-182}$	*HERC2/OCA2*
rs369230	G	113329	4.66 (4.45; 4.88)	0.02	$1.05x10^{-905}$	1.59 (1.56; 1.62)	0.01	$1.63x10^{-500}$	*MC1R*
rs6059655	A	117957	1.80 (1.70; 1.90)	0.03	$2.33x10^{-93}$	1.69 (1.65; 1.74)	0.01	$6.08x10^{-307}$	*RALY/ASIP*
rs11703668	G	117046	0.99 (0.95; 1.03)	0.02	0.617	0.93 (0.91; 0.95)	0.01	$4.76x10^{-17}$	*KIAA0930*

Supplementary Table 18. Summary statistics of associations with natural hair colour for known MC1R variants. For each variant, we report the effect allele, the effect size (OR/BETA) along with its 95% confidence interval (CI) and standard error (SE), the association P value in the UKBB study sample for red versus non-red hair colour (RH) and for non red-hair colour (non-RH). Positive OR_{RH} represent an increased incidence of red hair; positive $BETA_{non\text{-}RH}$ represent an increase in hair darkness.

Variant	SNP	Effect Allele	OR_{RH} (95% CI)	SE_{RH}	P_{RH}	$BETA_{non\text{-}RH}$ (95% CI)	$SE_{non\text{-}RH}$	$P_{non\text{-}RH}$
D84E	rs1805006	A	2.66 (2.36; 3.00)	0.06	$1.16x10^{-56}$	-0.153 (-0.178; -0.127)	0.013	$6.55x10^{-32}$
D294H	rs1805009	C	5.84 (5.43; 6.28)	0.04	$1.74x10^{-487}$	-0.128 (-0.147; -0.109)	0.010	$1.80x10^{-40}$
I155T	rs1110400	C	1.00 (0.82;1.21)	0.10	0.963	-0.096 (-0.123; -0.070)	0.013	$4.62x10^{-13}$
R142H	rs11547464	A	4.03 (3.51; 4.64)	0.07	$3.56x10^{-85}$	-0.104 (-0.138; -0.070)	0.018	$2.25x10^{-9}$
R151C	rs1805007	T	10.67 (10.10; 11.27)	0.03	$4.89x10^{-1529}$	-0.195 (-0.205; -0.185)	0.005	$4.05*10^{-310}$
R160W	rs1805008	T	3.73 (3.54; 3.93)	0.03	$8.66x10^{-348}$	-0.156 (-0.167; -0.146)	0.005	$7.54x10^{-197}$
R163Q	rs885479	A	0.14 (0.12; 0.17)	0.12	$1.77x10^{-59}$	-0.007 (-0.019; 0.006)	0.007	0.310
V60L	rs1805005	T	0.29 (0.26; 0.32)	0.05	$9.16x10^{-125}$	-0.044 (-0.053; -0.036)	0.004	$5.20x10^{-26}$
V92M	rs2228479	A	0.097 (0.08; 0.12)	0.10	$2.79x10^{-125}$	0.0285 (0.0194; 0.038)	0.005	$9.77x10^{-10}$

Supplementary Table 19. Summary statistics of associations with ease of skin tanning for known MC1R variants. For each variant, we report the effect allele, the minor allele frequency (MAF), the odds ratio (OR) along with its 95% confidence interval (CI) and standard error (SE), the association P value in the UKBB study sample. Positive ORs represent a decreased tanning ability.

Variant	SNP	Effect Allele	MAF	OR (95% CI)	SE	P
D84E	rs1805006	A	0.013	2.05 (1.90; 2.20)	0.04	7.06×10^{-82}
D294H	rs1805009	C	0.026	2.24 (2.13; 2.36)	0.03	5.86×10^{-204}
I155T	rs1110400	C	0.011	1.44 (1.33; 1.55)	0.04	2.71×10^{-20}
R142H	rs11547464	A	0.007	1.70 (1.54; 1.86)	0.05	7.15×10^{-28}
R151C	rs1805007	T	0.100	2.55 (2.48; 2.62)	0.01	8.71×10^{-907}
R160W	rs1805008	T	0.087	1.91 (1.86- 1.97)	0.02	2.72×10^{-403}
R163Q	rs885479	A	0.048	0.95 (0.91; 0.99)	0.02	8.08×10^{-3}
V60L	rs1805005	T	0.126	1.08 (1.05; 1.11)	0.01	2.450×10^{-9}
V92M	rs2228479	A	0.098	1.05 (1.02; 1.07)	0.01	2.08×10^{-3}

Supplementary Table 20. **cis-eQTL enrichment in skin tissues from the GTEx project.** Using the replicated SNPs (both primary and secondary associations) extended with any SNP in high linkage disequilibrium ($r^2 \geq 0.8$, N=599) with them, we evaluated empirical enrichment P values (eP) by comparing the overlap between the set of *cis*-eQTLs in the GTEx project database with this extended set of SNPs and with the overlap obtained using 1,000 random sets of SNPs.

**Tissue**	**eP**
Skin (fibroblasts)	1.0×10^{-3}
Sun exposed skin (Low leg)	1.0×10^{-3}
Non-sun exposed skin (Suprapubic)	0.142

Supplementary Table 21. **Enrichment for histone markers in epithelial foreskin melanocyte primary cells from the RoadMap**. Using the replicated SNPs (both primary and secondary associations) extended with any SNP in high linkage disequilibrium ($r^2 \geq 0.8$; N=599) with them, we evaluated empirical enrichment P values (eP) by comparing the overlap between DNA accessibility peaks and histone marks data from the Roadmap project with this extended set of SNPs and with the overlap obtained using 1000 random sets of SNPs. Since the histone modifications data for the studied cell line was available from two donors we averaged the overlaps among samples.

**Histone Mark**	**eP**
DNase	1.0×10^{-3}
H3K27ac	1.0×10^{-3}
H3K27me3	0.076
H3K36me3	2.0×10^{-3}
H3K4me1	1.0×10^{-3}
H3K4me3	3.0×10^{-3}
H3K9me3	0.066

Supplementary Notes

The Melanoma Meta-analysis Consortium

Law MH[1], Bishop DT[2], Lee JE[3], Brossard M[4,5], Moses EK[6], Song F[7], Barrett JH[2], Kumar R[8], Easton DF[9], Pharoah PD[10], Swerdlow AJ[11,12], Kypreou KP[13], Taylor JC[2], Harland M[2], Randerson-Moor J[2], Akslen LA[14,15], Andresen PA[16], Avril MF[17], Azizi E[18,19], Scarrà GB[20,21], Brown KM[22], Dębniak T[23], Elder DE[24], Fang S[3], Friedman E[19], Galan P[25], Ghiorzo P[20,21], Gillanders EM[26], Goldstein AM[22], Gruis NA[27], Hansson J[28], Helsing P[29], Hočevar M[30], Höiom V[28], Ingvar C[31], Chen WV[32]; GenoMEL Consortium; Essen-Heidelberg Investigators; SDH Study Group; Q-MEGA and QTWIN Investigators; AMFS Investigators; ATHENS Melanoma Study Group, Landi MT[22], Lang J[33], Lathrop GM[34], Lubiński J[23], Mackie RM[33,35], Mann GJ[36], Molven A[15,37], Montgomery GW[38], Novaković S[39], Olsson H[40,41], Puig S[42,43], Puig-Butille JA[42,43], LI X[44,45], Radford-Smith GL[46,47,48], van der Stoep N[49], van Doorn R[27], Whiteman DC[50], Craig JE[51], Schadendorf D[52,53], Simms LA[45], Burdon KP[54], Nyholt DR[38,55], Pooley KA[9], Orr N[56], Stratigos AJ[13], Cust AE[57], Ward SV[6], Hayward NK[58], Schulze HJ[59], Dunning AM[10], Bishop JA[2], Amos CI[60], MacGregor S[1]

[1]Statistical Genetics, QIMR Berghofer Medical Research Institute, Brisbane, Queensland, Australia.
[2]Section of Epidemiology and Biostatistics, Leeds Institute of Cancer and Pathology, University of Leeds, Leeds, UK.
[3]Department of Surgical Oncology, University of Texas MD Anderson Cancer Center, Houston, Texas, USA.
[4]INSERM, UMR 946, Genetic Variation and Human Diseases Unit, Paris, France.
[5]Institut Universitaire d'Hématologie, Université Paris Diderot, Sorbonne Paris Cité, Paris, France.
[6]Centre for Genetic Origins of Health and Disease, Faculty of Medicine, Dentistry and Health Sciences, University of Western Australia, Perth, Western Australia, Australia.
[7]Department of Epidemiology and Biostatistics, Key Laboratory of Cancer Prevention and Therapy, Tianjin, National Clinical Research Center of Cancer, Tianjin Medical University Cancer Institute and Hospital, Tianjin, China.
[8]Division of Molecular Genetic Epidemiology, German Cancer Research Center, Heidelberg, Germany.
[9]Centre for Cancer Genetic Epidemiology, Department of Public Health and Primary Care, University of Cambridge, Cambridge, UK.
[10]Centre for Cancer Genetic Epidemiology, Department of Oncology, University of Cambridge, Cambridge, UK.
[11]Division of Genetics and Epidemiology, The Institute of Cancer Research, London, UK.
[12]Division of Breast Cancer Research, The Institute of Cancer Research, London, UK.
[13]Department of Dermatology, University of Athens School of Medicine, Andreas Sygros Hospital, Athens, Greece.
[14]Centre for Cancer Biomarkers (CCBIO), Department of Clinical Medicine, University of Bergen, Bergen, Norway.
[15]Department of Pathology, Haukeland University Hospital, Bergen, Norway.
[16]Department of Pathology, Molecular Pathology, Oslo University Hospital, Rikshospitalet, Oslo, Norway.
[17]Assistance Publique-Hôpitaux de Paris, Hôpital Cochin, Service de Dermatologie, Université Paris Descartes, Paris, France.
[18]Department of Dermatology, Sheba Medical Center, Tel Hashomer, Sackler Faculty of Medicine, Tel Aviv, Israel.
[19]Oncogenetics Unit, Sheba Medical Center, Tel Hashomer, Sackler Faculty of Medicine, Tel Aviv University, Tel Aviv, Israel.
[20]Department of Internal Medicine and Medical Specialties, University of Genoa, Genoa, Italy.
[21]Laboratory of Genetics of Rare Cancers, Istituto di Ricovero e Cura a Carattere Scientifico Azienda Ospedaliera Universitaria (IRCCS AOU) San Martino l'Istituto Scientifico Tumori Istituto Nazionale per la Ricerca sul Cancro, Genoa, Italy.

[22]Division of Cancer Epidemiology and Genetics, National Cancer Institute, US National Institutes of Health, Bethesda, Maryland, USA.
[23]International Hereditary Cancer Center, Pomeranian Medical University, Szczecin, Poland.
[24]Department of Pathology and Laboratory Medicine, Perelman School of Medicine at the University of Pennsylvania, Philadelphia, Pennsylvania, USA.
[25]Université Paris 13, Equipe de Recherche en Epidémiologie Nutritionnelle (EREN), Centre de Recherche en Epidémiologie et Statistiques, INSERM U1153, Institut National de la Recherche Agronomique (INRA) U1125, Conservatoire National des Arts et Métiers, Communauté d'Université Sorbonne Paris Cité, Bobigny, France.
[26]Inherited Disease Research Branch, National Human Genome Research Institute, US National Institutes of Health, Baltimore, Maryland, USA.
[27]Department of Dermatology, Leiden University Medical Center, Leiden, the Netherlands.
[28]Department of Oncology-Pathology, Karolinska Institutet, Karolinska University Hospital, Stockholm, Sweden.
[29]Department of Dermatology, Oslo University Hospital, Rikshospitalet, Oslo, Norway.
[30]Department of Surgical Oncology, Institute of Oncology Ljubljana, Ljubljana, Slovenia.
[31]Department of Surgery, Clinical Sciences, Lund University, Lund, Sweden.
[32]Department of Genetics, University of Texas MD Anderson Cancer Center, Houston, Texas, USA.
[33]Department of Medical Genetics, University of Glasgow, Glasgow, UK.
[34]McGill University and Génome Québec Innovation Centre, Montreal, Quebec, Canada.
[35]Department of Public Health, University of Glasgow, Glasgow, UK.
[36]Centre for Cancer Research, University of Sydney at Westmead, Millennium Institute for Medical Research and Melanoma Institute Australia, Sydney, New South Wales, Australia.
[37]Gade Laboratory for Pathology, Department of Clinical Medicine, University of Bergen, Bergen, Norway.
[38]Molecular Epidemiology, QIMR Berghofer Medical Research Institute, Brisbane, Queensland, Australia.
[39]Department of Molecular Diagnostics, Institute of Oncology Ljubljana, Ljubljana, Slovenia.
[40]Department of Oncology/Pathology, Clinical Sciences, Lund University, Lund, Sweden.
[41]Department of Cancer Epidemiology, Clinical Sciences, Lund University, Lund, Sweden.
[42]Melanoma Unit, Departments of Dermatology, Biochemistry and Molecular Genetics, Hospital Clinic, Institut d'Investigacions Biomèdica August Pi Suñe, Universitat de Barcelona, Barcelona, Spain.
[43]Centro de Investigación Biomédica en Red (CIBER) de Enfermedades Raras, Instituto de Salud Carlos III, Barcelona, Spain.
[44]Department of Epidemiology, Richard M. Fairbanks School of Public Health, Indiana University, Indianapolis, Indiana, USA.
[45]Melvin and Bren Simon Cancer Center, Indiana University, Indianapolis, Indiana, USA.
[46]Inflammatory Bowel Diseases, QIMR Berghofer Medical Research Institute, Brisbane, Queensland, Australia.
[47]Department of Gastroenterology and Hepatology, Royal Brisbane and Women's Hospital, Brisbane, Queensland, Australia.
[48]University of Queensland School of Medicine, Herston Campus, Brisbane, Queensland, Australia.
[49]Department of Clinical Genetics, Center of Human and Clinical Genetics, Leiden University Medical Center, Leiden, the Netherlands.
[50]Cancer Control Group, QIMR Berghofer Medical Research Institute, Brisbane, Queensland, Australia.
[51]Department of Ophthalmology, Flinders University, Adelaide, South Australia, Australia.
[52]Department of Dermatology, University Hospital Essen, Essen, Germany.
[53]German Consortium for Translational Cancer Research (DKTK), Heidelberg, Germany.
[54]Menzies Institute for Medical Research, University of Tasmania, Hobart, Tasmania, Australia.
[55]Institute of Health and Biomedical Innovation, Queensland University of Technology, Brisbane, Queensland, Australia.
[56]Breakthrough Breast Cancer Research Centre, The Institute of Cancer Research, London, UK.
[57]Cancer Epidemiology and Services Research, Sydney School of Public Health, University of Sydney, Sydney, New South Wales, Australia.

[58]Oncogenomics, QIMR Berghofer Medical Research Institute, Brisbane, Queensland, Australia.
[59]Department of Dermatology, Fachklinik Hornheide, Institute for Tumors of the Skin at the University of Münster, Münster, Germany.
[60]Department of Community and Family Medicine, Geisel School of Medicine, Dartmouth College, Hanover, New Hampshire, USA.
