# Supplementary material for: Genome-wide association study in 176,678 Europeans reveals genetic loci for tanning response to sun exposure
Source: Nat Commun. 2018 May 8;9:1684. doi: 10.1038/s41467-018-04086-y (PMC5940788; doi:10.1038/s41467-018-04086-y)
Supplement: Supplementary file 3 — Description of Additional Supplementary Files [file 41467_2018_4086_MOESM3_ESM.pdf]

Description of Additional Supplementary Files

File Name: Supplementary Data 1
Description: The association summary statistics for the 10,834 genome-wide significant SNPs are provided as Supplementary Data 1.

File Name: Supplementary Data 2
Description: Genomic coordinates are reported in GRCh37.p13. The association summary statistics for the SNPs associated in the UKBB non-melanoma skin cancer GWAS (P < 1x10-5) are provided as Supplementary Data 2.

File Name: Supplementary Data 3
Description: Genomic coordinates are reported in GRCh37.p13. The ciseQTLs identified in the three studied skin tissues, and available within the GTEx project, are provided as Supplementary Data 3.
